# Diagnostic Yield of Population-Based Screening for Chronic Kidney Disease in Low-Income, Middle-Income, and High-Income Countries

**DOI:** 10.1001/jamanetworkopen.2021.27396

**Published:** 2021-10-04

**Authors:** Marcello Tonelli, Sophanny Tiv, Shuchi Anand, Deepa Mohan, Guillermo Garcia Garcia, José Alfonso Gutiérrez Padilla, Scott Klarenbach, Guillermo Navarro Blackaller, Sidy Seck, Jinwei Wang, Luxia Zhang, Paul Muntner

**Affiliations:** 1 University of Calgary, Calgary, Alberta, Canada; 2 Division of Nephrology, Department of Medicine, University of Alberta, Edmonton, Alberta, Canada; 3 Division of Nephrology, Department of Medicine, Stanford University, Stanford, California; 4 Madras Diabetes Research Foundation, Chennai, India; 5 Hospital Civil de Guadalajara Fray Antonio Alcalde, University Center for Health Science, University of Guadalajara, Guadalajara, Jalisco, Mexico; 6 Department of Internal Medicine and Nephrology, Gaston Berger University, Saint-Louis, Senegal; 7 Peking University Institute of Nephrology, Peking University First Hospital, Beijing, China; 8 Department of Epidemiology, School of Public Health, University of Alabama, Tuscaloosa

## Abstract

**Importance:**

Population-based screening for chronic kidney disease (CKD) is sometimes recommended based on the assumption that detecting CKD is associated with beneficial changes in treatment. However, the treatment of CKD is often similar to the treatment of hypertension or diabetes, which commonly coexist with CKD.

**Objective:**

To determine the frequency with which population-based screening for CKD is associated with a change in recommended treatment compared with a strategy of measuring blood pressure and assessing glycemia.

**Design, Setting, and Participants:**

This cohort study was conducted using data obtained from studies that evaluated CKD in population-based samples from China (2007-2010), India (2010-2014), Mexico (2007-2008), Senegal (2012), and the United States (2009-2014), including a total of 126 242 adults screened for CKD. Data were analyzed from January 2020 to March 2021.

**Main Outcomes and Measures:**

The primary definition of CKD was estimated glomerular filtration rate less than 60 mL/min/1.73 m^2^. For individuals with CKD, the need for a treatment change was defined as not taking an angiotensin-converting enzyme inhibitor or angiotensin receptor blocker or having blood pressure levels of 140/90 mm Hg or greater. For individuals with CKD who also had diabetes, the need for a treatment change was also defined as having hemoglobin A_1c_ levels of 8% or greater or fasting glucose levels of 178.4 mg/dL (9.9 mmol/L) or greater. Case finding was defined as testing for CKD only in adults with hypertension or diabetes.

**Results:**

Among 126 242 adults screened for CKD, there were 47 204 patients in the China cohort, 9817 patients in the India cohort, 51 137 patients in the Mexico cohort, 2441 patients in the Senegal cohort, and 15 643 patients in the US cohort. The mean age of participants was 49.6 years (95% CI, 49.5-49.7 years) in the China cohort, 42.9 years (95% CI, 42.6-43.2 years) in the India cohort, 51.6 years (95% CI, 51.5-51.7 years) in the Mexico cohort, 48.2 years (95% CI, 47.5-48.9 years) in the Senegal cohort, and 47.3 years (95% CI, 46.6-48.0 years) in the US cohort. The proportion of women was 57.3% (95% CI, 56.9%-57.7%) in the China cohort, 53.4% (95% CI, 52.4%-54.4%) in the India cohort, 68.8% (95% CI, 68.4%-69.2%) in the Mexico cohort, 56.0% (95% CI, 54.0%-58.0%) in the Senegal cohort, and 51.9% (51.0%-52.7%) in the US cohort. The prevalence of CKD was 2.5% (95% CI, 2.4%-2.7%) in the China cohort, 2.3% (95% CI, 2.0%-2.6%) in the India cohort, 10.6% (95% CI, 10.3%-10.9%) in the Mexico cohort, 13.1% (95% CI, 11.7%-14.4%) in the Senegal cohort, and 6.8% (95% CI, 6.2%-7.5%) in the US cohort. Screening for CKD was associated with the identification of additional adults whose treatment would change (beyond those identified by measuring blood pressure and glycemia) per 1000 adults: China: 8 adults (95% CI, 8-9 adults); India: 5 adults (95% CI, 4-7 adults); Mexico: 26 adults (95% CI, 24-27 adults); Senegal: 59 adults (95% CI, 50-69 adults); and the US: 19 adults (95% CI, 16-23 adults). Case finding was associated with the identification of 46.2% (95% CI, 45.1%-47.4%) to 86.4% (95% CI, 85.4%-87.3%) of individuals with CKD depending on the country, an increase in the proportion of individuals requiring a treatment change by as much 89.6% (95% CI, 80.4%-99.3%) in the US, and a decrease in the proportion of individuals needing GFR measurements by as much as 57.8% (95% CI, 56.3%-59.3%) in the US.

**Conclusions and Relevance:**

This study found that most additional individuals with CKD identified by population-based screening programs did not need a change in treatment compared with a strategy of measuring blood pressure and assessing glycemia and that case finding was more efficient than screening for early detection of CKD.

## Key Points

**Question:**

How frequently is population-based screening for chronic kidney disease (CKD) associated with a change in recommended treatment compared with a strategy of measuring blood pressure and assessing glycemia?

**Findings:**

This epidemiologic assessment of 126 242 adults screened for CKD in population-based cohorts from China, India, Mexico, Senegal, and the United States found that most treatment gaps identified by population-based screening for CKD were apparent by measuring blood pressure or glycemic control. Case finding, defined by testing for CKD only in adults with hypertension or diabetes, was associated with a lower frequency of testing and a greater proportion of individuals with identified treatment gaps compared with screening.

**Meaning:**

These findings suggest that case finding was more efficient than population-based screening and detected most patients with CKD requiring treatment changes.

## Teaser

This epidemiologic assessment of population-based cohorts investigates the change in treatment for chronic kidney disease associated with population-based screening compared with measuring blood pressure and assessing glycemia.

## Introduction

Chronic kidney disease (CKD) is a common condition that is associated with substantial morbidity and mortality.^1^ The prevalence and burden of CKD are increasing rapidly in low- and middle-income countries (LMICs),^2,3^ which has been associated with increasing demand for expensive kidney replacement. Most individuals in LMICs cannot afford kidney replacement,^4^ and CKD that progresses to kidney failure kills millions of people each year.^5^ Because widely available and inexpensive treatments can slow or prevent loss of kidney function and CKD is asymptomatic until its later stages, some have advocated for population-based screening to enable early intervention in LMICs and high-income countries.^6^

In theory, screening for CKD would allow for earlier initiation of treatment, delaying or avoiding progression to kidney failure. Treatments that are known to prevent progressive loss of kidney function in CKD include control of blood pressure, use of an angiotensin-converting enzyme inhibitor (ACEI) or angiotensin receptor blocker (ARB), and control of blood glucose among individuals with diabetes.^7,8,9,10,11,12,13^ Sodium-glucose cotransporter-2 (SGLT2) inhibitors also improve outcomes in individuals with CKD,^14,15^ although these medications are not widely used at present, especially in LMICs. For a small proportion (<10%) of individuals with CKD, early detection is associated with the identification of an underlying condition, such as glomerulonephritis, and additional treatment.^16,17^ Any incremental benefit associated with screening occurs during the period between the detection of CKD by screening and when it would otherwise be detected. However, this assumes that earlier detection is associated with beneficial changes in treatment. This assumption is uncertain, given the overlap in the recommended treatment between CKD and other noncommunicable diseases (NCDs).^17,18,19^

We designed the current study to estimate the yield associated with population-based screening for CKD in low-income, middle-income, and high-income settings, including China, India, Mexico, Senegal, and the United States. We hypothesized that most instances in which CKD is detected by population-based screening would not necessarily be associated with a change in treatment. We also hypothesized that testing for CKD among individuals with a self-reported history of hypertension, diabetes, or CKD and those with current evidence of hypertension or diabetes (ie, individuals tested in case finding) would be associated with an increase in the proportion of individuals with detected CKD who would be recommended a treatment change and a significant decrease in the number of individuals who required testing for CKD.

## Methods

This epidemiologic assessment of population-based cohorts was approved by the Health Research Ethics Board at the University of Calgary. Informed consent was obtained from participants in the original cohort studies.

Data were obtained from 5 previously completed studies, each of which evaluated the prevalence of CKD in a population-based sample from 1 of the countries of interest: China,^20^ India,^21^ Mexico,^22^ Senegal,^23^ or the United States.^24^ All studies measured kidney function with estimated glomerular filtration rate (eGFR) or albuminuria on a single occasion. The Mexico and Senegal studies excluded participants receiving kidney replacement, whereas the others did not. The Mexico study also excluded individuals who reported that they had CKD, whereas the other studies did not. Details of each cohort are presented in the eMethods in the Supplement. We performed a complete case analysis.

### Definitions of CKD

The primary definition of CKD was based on eGFR less than 60 mL/min/1.73 m^2^ at the time of the study visit; a second measure more than 3 months from the initial measure was not required. In sensitivity analyses, we considered 2 alternative definitions of CKD: (1) eGFR less than 60 mL/min/1.73 m^2^ or severe albuminuria^1^ and (2) eGFR less than 45 mL/min/1.73 m^2^ with or without severe albuminuria.^2^ Severe albuminuria was defined as the presence of any 1 of the following: albumin-to-creatinine ratio greater than 300 mg/g or greater than 30 mg/mmol; protein-to-creatinine ratio greater than 500 mg/g or greater than 50 mg/mmol; dipstick urinalysis of 2 or greater for albumin. We focused on severe rather than moderate albuminuria because it is associated with worse prognosis, may be less likely to be a false positive finding, and is more readily detected using dipstick urine testing, which is especially relevant for LMICs.

### Definitions of Need for Treatment Change

A history of diabetes, CKD, or hypertension was based on self-report and definitions used by each study (eMethods in the Supplement) Data on use of antihypertensive medication were obtained for the India, Senegal, and US cohorts. Use of ACEI or ARB was defined by self-report or review of medication records showing that 1 or more of these medications were currently being taken; we did not have data to evaluate the doses of these medications or assess whether an increase in the dose of ACEI or ARB was indicated. Because data on medication use were not available for the China and Mexico cohorts, we used the proportion of ACEI or ARB use from the US cohort to estimate medication use in these cohorts. We tested the association of this extrapolation with outcomes in sensitivity analyses that (1) decreased the proportion of ACEI or ARB use in each country by 10% compared with the proportion in the United States and (2) similarly increased it by 10%.

We defined appropriate blood pressure control as less than 140/90 mm Hg^25^ using measurements from each study; participants with appropriate blood pressure control were not considered to require a change to their blood pressure treatment. We selected a target of less than 140/90 mm Hg given that it is conservative and will tend to overestimate the benefits associated with early detection of CKD compared with a target of less than 130/80 mm Hg.^26^

Appropriate glycemic control was defined by hemoglobin A_1c_ (HbA_1c_) levels of less than 8% (0.08 of total hemoglobin)^27^ in the India and US cohorts and by fasting blood glucose levels of less than 178.4 mg/dL (9.9 mmol/L) based on a published conversion factor^19^ for the Mexico, Senegal, and China cohorts given that HbA_1c_ data were not available. Participants with appropriate glycemic control were considered not to require any change to their glycemic treatment. We chose a threshold of less than 8% because it will tend to overestimate the benefits of early detection compared with an individualized threshold of less than 6.5% (0.065 of total hemoglobin) to 8%.^27^

For participants with CKD but not diabetes, the need for a treatment change was defined as not taking an ACEI or ARB or having blood pressure levels of 140/90 mm Hg or more. For participants with CKD and diabetes, the need for a treatment change was defined as not taking an ACEI or ARB, having blood pressure levels of 140/90 mm Hg or more, having HbA_1c_ levels of 8% (0.08 of total hemoglobin) or more in the India and United States cohorts, or having fasting glucose levels of 178.4 mg/dL (9.9 mmol/L) or more in the Mexico, Senegal, and China cohorts. Given that taking an ACEI or ARB may not be associated with a benefit among individuals with CKD and well-controlled albuminuria but without diabetes, this definition may be associated with overestimated benefits of early detection.

We assumed that detecting hypertension suggested the need to control blood pressure and that first-line treatment for individuals with CKD and hypertension would be an ACEI or ARB.^18^ We also assumed that detecting uncontrolled glycemia required a treatment change. Therefore, the situation in which a measurement of kidney function would influence treatment decisions was defined by the identification of CKD in a participant without hypertension or diabetes who was not receiving an ACEI or ARB.

### Definitions of Screening vs Case Finding

For the primary definition of CKD (ie, eGFR < 60 mL/min/1.73 m^2^) and the definition of CKD used in the second sensitivity analysis (ie, eGFR < 45 mL/min/1.73 m^2^), screening was defined as measuring eGFR in all participants. In the definition of CKD used in the first sensitivity analysis (ie, eGFR < 60 mL/min/1.73 m^2^ or severe albuminuria), screening included measuring eGFR and albuminuria. Case finding was defined by measuring eGFR in the subset of participants with a self-reported history of hypertension, diabetes, or CKD or with blood pressure levels of 140/90 mm Hg or more or with laboratory evidence of diabetes, HbA_1c_ levels of 6.5% (0.065 of total hemoglobin)) or more, or fasting blood glucose levels of 126.1 mg/dL (7.0 mmol/L) or more depending on cohort.

### Statistical Analysis

For each study, information on participant characteristics; prevalence of diabetes, hypertension, and CKD; blood pressure and glycemia control status; and ACEI and ARB use were estimated and recorded in a standardized form. Results were checked (S.T.) and expressed as No. (%) with 95% CI. When evaluating population-based screening, we calculated the proportion of individuals in the overall population who required a treatment change, then stratified these results by whether detecting the need for a treatment change required knowledge of CKD status. We calculated the proportion of individuals with CKD detected by population-based screening and categorized this population into 3 groups^1^: those for whom no change in treatment was needed,^2^ those for whom a change in treatment was needed without assessing CKD status, and^3^ those who required a change in treatment that was based on knowledge of CKD status. We further subdivided these 3 groups of individuals with CKD into 3 strata^1^: those with hypertension without diabetes,^2^ those with diabetes with or without hypertension, and^3^ those without hypertension or diabetes. We converted proportions of individuals needing a treatment change to the number per 1000 individuals screened. The number needed to screen (NNS) to identify 1 individual for whom a treatment change was required was calculated as 1 divided by the percentage of individuals for whom a treatment change was required. The data were expressed in tabular form and as Senn plots and stacked bar graphs.

We then calculated the number of individuals for whom the assessment of CKD status would be required using the case-finding approach. Additionally, we calculated the number and proportion of individuals with CKD identified and the proportion of those identified for whom a treatment change would be required through case finding using the same methods as those used to evaluate population-based screening described previously. We calculated the percentage reduction in number of individuals who would have eGFR measurements and the increase in the proportion of individuals with CKD identified who required a treatment change for the case-finding vs population-wide screening approaches. Additionally, we calculated the proportion of individuals with CKD in each cohort identified by case finding. Data were reported as means or percentages with 95% CIs calculated using Excel 2019 (Microsoft Corporation). Data were analyzed from January 2020 to March 2021.

## Results

Among 126 242 adults screened for CKD, there were 47 204 patients in the China cohort, 9817 patients in the India cohort, 51 137 patients in the Mexico cohort, 2441 patients in the Senegal cohort, and 15 643 patients in the US cohort. The mean age of participants was 49.6 years (95% CI, 49.5-49.7 years) in the China cohort, 42.9 years (95% CI, 42.6-43.2 years) in the India cohort, 51.6 years (95% CI, 51.5-51.7 years) in the Mexico cohort, 48.2 years (95% CI, 47.5-48.9 years) in the Senegal cohort, and 47.3 years (95% CI, 46.6-48.0 years) in the US cohort. The proportion of women was 57.3% (95% CI, 56.9%-57.7%) in the China cohort, 53.4% (95% CI, 52.4%-54.4%) in the India cohort, 68.8% (95% CI, 68.4%-69.2%) in the Mexico cohort, 56.0% (95% CI, 54.0%-58.0%) in the Senegal cohort, and 51.9% (95% CI, 51.0%-52.7%) in the US cohort (Table 1).

**Table 1.** Characteristics of Participants

Characteristic	Proportion of study cohort, % (95% CI)				
	China (n = 47 204)	India (n = 9817)	Mexico (n = 51 137)	Senegal (n = 2441)	United States (n = 15 643^a^)
Age, mean (95% CI), y	49.6 (49.5-49.7)	42.9 (42.6-43.2)	51.6 (51.5-51.7)	48.2 (47.5-48.9)	47.3 (46.6-48.0)
Sex					
Women	57.3 (56.9-57.8)	53.4 (52.4-54.4)	68.8 (68.4-69.2)	56.0 (54.0-58.0)	51.9 (51.0-52.7)
Men	42.7 (42.2-43.1)	46.6 (45.6-47.6)	31.2 (30.8-31.6)	44.0 (42.0-46.0)	48.1 (47.2-49.0)
Tobacco use	23.5 (23.1-23.9)	22.5 (21.7-23.3)	18.8 (18.5-19.1)	4.5 (3.7-5.3)	19.8 (18.7-21.0)
Self-reported history of hypertension	20.1 (19.7-20.5)	14.2 (13.5-14.9)	32.7 (32.3-33.1)	33.9 (32.0-35.8)	32.9 (31.5-34.4)
Self-reported history of diabetes	5.1 (4.9-5.3)	13.0 (12.3-13.7)	22.5 (22.1-22.9)	26.4 (24.7-28.1)	11.1 (10.4-11.7)
Self-reported history of CKD	5.2 (5.0-5.4)	0.2 (0.1-0.3)	0 (0-1.0)	4.9 (4.0-5.8)	2.3 (2.0-2.6)
Receiving ACEI or ARB	NA	3.1 (2.8-3.4)	NA	18.9 (17.3-20.5)	18.0 (16.8-19.3)
BMI, mean (95% CI)	23.9 (23.9-23.9)	25.6 (25.5-25.7)	28.5 (28.5-28.5)	24.3 (24.1-24.5)	28.8 (28.6-29.0)
Systolic blood pressure, mean (95% CI), mmHg	126.9 (126.7-127.1)	123.6 (123.2-124.0)	136.6 (136.4-136.8)	131.0 (130.1-131.9)	121.5 (120.9-122.0)
Diastolic blood pressure, mean (95% CI), mmHg	80.1 (80.0-80.2)	82.0 (81.8-82.2)	80.2 (80.1-80.3)	86.3 (85.6-87.0)	70.6 (70.1-71.2)
HbA_1c_, mean (95% CI), %	NA	6.3 (6.3-6.3)	NA	NA	5.6 (5.6-5.7)
Fasting glucose, mean (95% CI), mg/dL	95.5 (95.2-95.8)	118.9 (117.9-119.9)	118.9 (118.4-119.4)	108.1 (105.7-110.5)	NA
eGFR, mean (95% CI), mL/min/1.73 m^2^	101.2 (101.0-101.4)	106.8 (106.4-107.2)	77.4 (77.2-77.6)	101.3 (100.4-102.2)	94.1 (93.3-94.9)
Severe albuminuria^b^	0.7 (0.6-0.7)	0.9 (0.7-1.1)	1.1 (1.0-1.2)	5.3 (4.4-6.2)	2.1 (1.9-2.3)
Prevalence of chronic kidney disease					
Patients with CKD, No.	1185	225	5413	319	1291
Known CKD^c^	14.2 (12.2-16.2)	6.2 (5.6-6.8)	0	6.3 (5.5-7.0)	13.2 (11.7-14.4)
Primary definition of CKD	2.5 (2.4-2.7)	2.3 (2.0-2.6)	10.6 (10.3-10.9)	13.1 (11.7-14.4)	6.8 (6.2-7.5)
Sensitivity analysis 1	3.0 (2.9-3.2)	2.9 (2.6-3.3)	11.3 (11.0-11.6)	14.8 (13.4-16.2)	8.1 (7.5-8.8)
Sensitivity analysis 2	0.5 (0.5-0.6)	0.8 (0.7-1.0)	2.0 (1.9-2.2)	5.2 (4.3-6.0)	2.2 (1.9-2.5)
Individuals with CKD requiring treatment change^d^					
Primary definition of CKD	89.9 (88.2-91.6)	97.8 (95.9-99.7)	86.8 (85.9-87.7)	78.7 (74.2-83.2)	68.7 (65.1-72.1)
Sensitivity analysis 1	90.6 (89.1-92.1)	95.1 (92.6-97.6)	87.2 (86.3-88.0)	77.3 (73.0-81.7)	71.5 (68.5-74.4)
Sensitivity analysis 2	87.8 (83.7-91.9)	96.3 (92.2-100)	85.4 (83.3-87.6)	73.8 (66.1-81.5)	65.6 (60.7-70.2)

Abbreviations: ACEI, angiotensin-converting enzyme inhibitor; ARB, angiotensin receptor blocker; BMI, body mass index (calculated as weight in kilograms divided by height in meters squared); CKD, chronic kidney disease; eGFR, estimated glomerular filtration rate; HbA_1c_, hemoglobin A_1c_; NA, not available.

SI conversion factors: To convert glucose to mmol/L, multiply by 0.0555; percent of total hemoglobin to proportion of total hemoglobin, multiply by 0.01.

^a^ Data for the United States are weighted to the US population as described in the eMethods section in the Supplement. The sample size represents 223 million US adults aged 20 years or older.

^b^ Severe albuminuria was defined by any 1 of an albumin-to-creatinine ratio greater than 300 mg/g or greater than 30 mg/mmol, a protein-to-creatinine ratio greater than 500 mg/g or greater than 50 mg/mmol, or a dipstick urinalysis result of 2 or greater for albumin.

^c^ The definition of CKD was eGFR less than 60 mL/min/1.73 m^2^ in the primary definition, eGFR less than 60 mL/min/1.73 m^2^ or severe albuminuria in sensitivity analysis 1, and eGFR < 45 mL/min/1.73 m^2^ in sensitivity analysis 2.

^d^ For participants with CKD but without diabetes, the need for a treatment change was defined by no use of ACEI or ARB or blood pressure above the target value of 140/90 mm Hg. For participants with CKD and diabetes, the need for a treatment change was defined by no use of ACEI or ARB, blood pressure above the target value of 140/90 mm Hg, HbA_1c_ levels of 8% (0.08 of total hemoglobin) or more, or fasting glucose levels of 178.4 mg/dL (9.9 mmol/L) or more.

**Figure 1.**
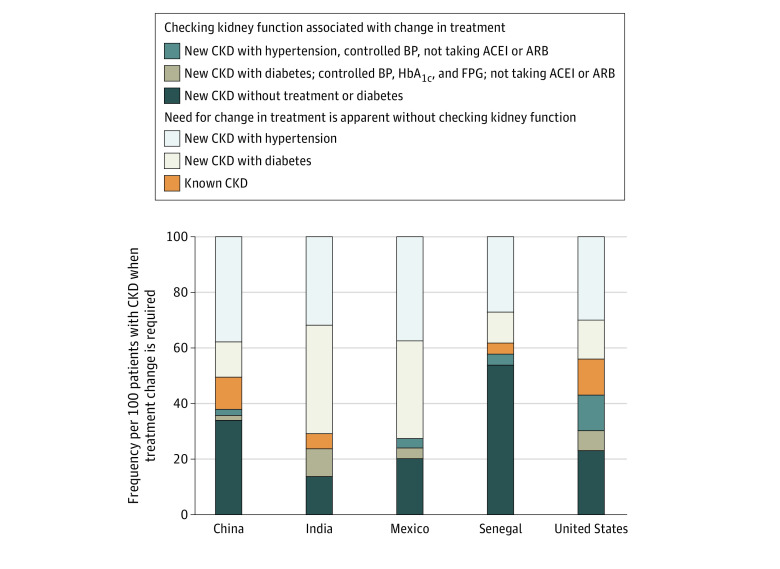
Indications for Changes in Treatment in Screen-detected Chronic Kidney Disease (CKD) The need for change in treatment was determined by the presence or absence of hypertension and diabetes or by CKD diagnoses, defined by estimated glomerular filtration rate less than 60 mL/min/1.73 m^2^. The new CKD with hypertension group includes only individuals without diabetes. The new CKD with diabetes group includes individuals with or without hypertension. ACEI indicates angiotensin-converting enzyme inhibitor; ARB, angiotensin receptor blocker; BP, blood pressure; FPG, fasting plasma glucose; HbA_1c_, hemoglobin A_1c_.

When assessed using the primary definition (ie, eGFR < 60 mL/min/1.73 m^2^), the prevalence of CKD was 2.5% (95% CI, 2.4%-2.7%) in the China cohort, 2.3% (95% CI, 2.0%-2.6%) in the India cohort, 10.6% (95% CI, 10.3%-10.9%) in the Mexico cohort, 13.1% (95% CI, 11.7%-14.4%) in the Senegal cohort, and 6.8% (95% CI, 6.2%-7.5%) in the US cohort. Among individuals in these cohorts with CKD, the proportion identified with CKD prior to screening (ie, those with known CKD) was 14.2% (95% CI, 12.2%-16.2%) in the China cohort, 6.2% (95% CI, 5.6%-6.8%) in the India cohort, 0% in the Mexico cohort, 6.3% (95% CI, 5.5%-7.0%) in the Senegal cohort, and 13.2% (95% CI, 11.1%-15.7%) in the US cohort. Among individuals with CKD, the proportion who required a treatment change ranged from 68.7% (95% CI, 65.1%-72.1%) in the US cohort to 97.8% (95% CI, 95.9%-99.7%) in the India cohort. However, with the exception of individuals in Senegal, most individuals with CKD who required a treatment change could be identified without checking kidney function by measuring blood pressure or testing for diabetes (Figure 1).

### Yield of Population-Based Screening

**Figure 2.**
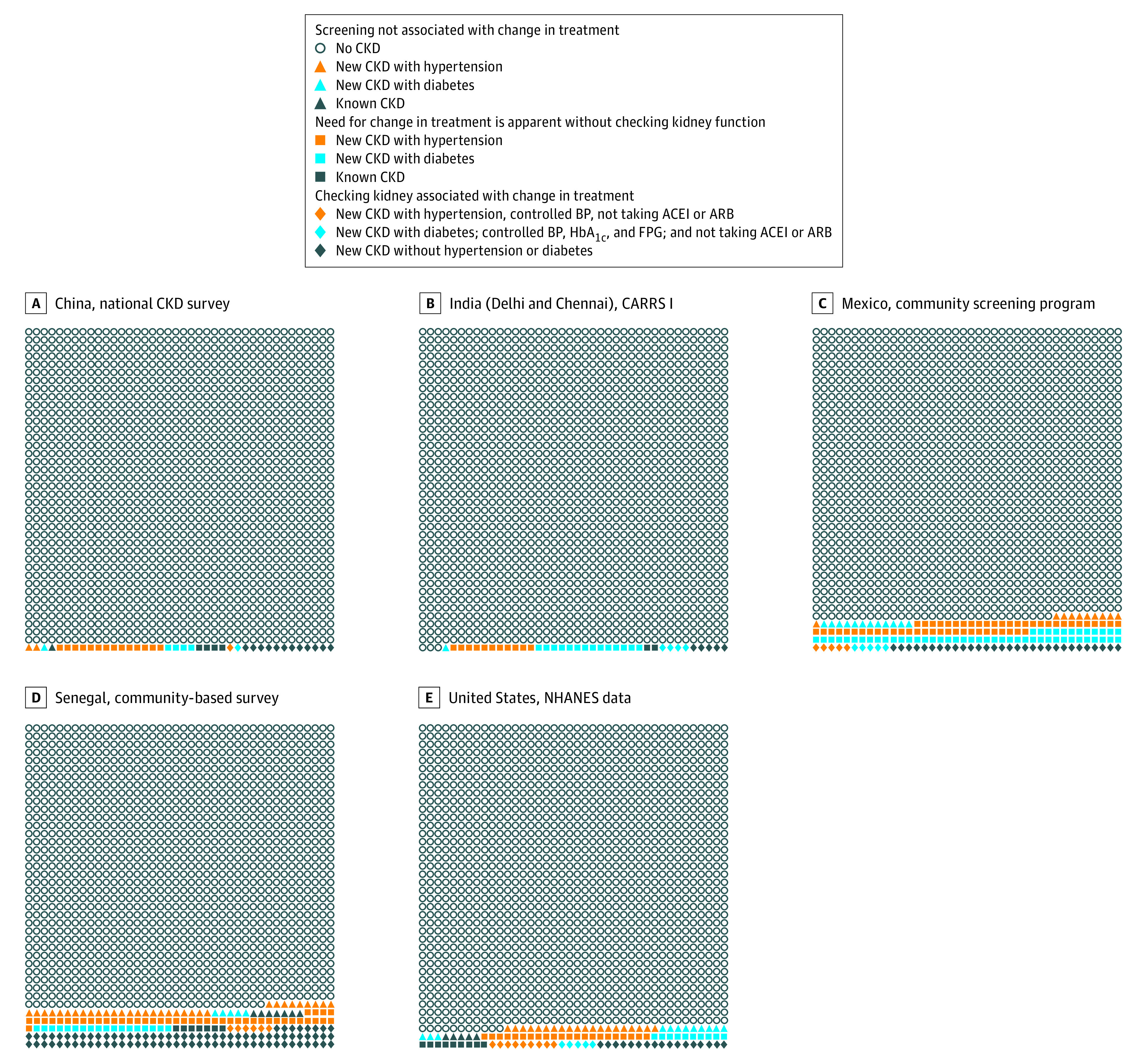
Yield of Screening for Chronic Kidney Disease (CKD) CKD was defined by the primary definition (ie, estimated glomerular filtration rate < 60 mL/min/1.73 m^2^). The figure graphically presents the number of individuals in each category, expressed per 1600 individuals. Data were based on Table 2. The new CKD with hypertension group includes only individuals without diabetes. The new CKD with diabetes group includes individuals with or without hypertension. ACEI indicates angiotensin-converting enzyme inhibitor; ARB, angiotensin receptor blocker; BP, blood pressure; CARRS, Centre for Cardio-metabolic Risk Reduction in South Asia; HbA_1c_, hemoglobin A_1c_; FPG, fasting plasma glucose; NHANES, National Health and Nutrition Examination Survey.

Assessing CKD status was associated with the identification of additional participants for whom treatment would change per 1000 participants: China: 8 adults (95% CI, 8 adults-9 adults); India: 5 adults (95% CI, 4 adults-7 adults); and the US: 19 adults (95% CI, 16 adults-23 adults) (Table 2). In the Mexico and Senegal cohorts, screening was associated with identification of a change in treatment for 26 additional adults (95% CI, 24 adults-27 adults) and 59 additional adults (95% CI, 50 adults-69 adults) per 1000 participants, respectively, by assessing CKD status (Table 2). The NNS to find 1 individual for whom assessing CKD status was associated with a change treatment were 117 individuals (95% CI, 107 individuals-130 individuals) for China, 189 individuals (95% CI, 149 individuals-259 individuals) for India, 40 individuals (95% CI, 38 individuals-42 individuals) for Mexico, 17 individuals (95% CI, 15 individuals-20 individuals) for Senegal, and 52 individuals (95% CI, 44 individuals-62 individuals) for the United States. Figure 2 shows a Senn plot of the yield of population-based screening.

**Table 2.** Yield of Screening for CKD

Group^a^	Patients per 1000 individuals screened, No. (95% CI)				
	China	India	Mexico	Senegal	United States	
**Primary definition**					
No CKD	975 (973-976)	977 (974-980)	895 (892-897)	869 (856-883)	932 (925-938)	
CKD but no change in treatment						
New CKD with hypertension^b^	1 (1-2)	0 (0-1)	6 (5-7)	21 (15-26)	13 (10-15)	
New CKD with diabetes^c^	0 (0-1)	1 (0-1)	7 (7-8)	3 (1-6)	8 (6-9)	
Known CKD	1 (1-2)	0 (0-1)	0 (0-1)	4 (2-7)	3 (2-4)	
Total	2 (2-3)	1 (0-1)	13 (12-14)	28 (21-34)	24 (20-27)	
Need for change in treatment without assessing eGFR or albuminuria						
New CKD with hypertension	9 (8-9)	7 (5-9)	34 (33-36)	28 (21-34)	13 (12-16)	
New CKD with diabetes	3 (2-3)	9 (7-11)	32 (31-34)	12 (7-16)	6 (5-8)	
Known CKD	3 (2-3)	1 (1-2)	0 (0-1)	4 (2-7)	6 (5-8)	
Total	15 (13-15)	17 (15-20)	66 (65-69)	44 (35-52)	25 (23-29)	
Need for change in treatment based on eGFR or albuminuria						
New CKD with hypertension, controlled BP, not taking ACEI or ARB	0 (0-1)	0 (0-1)	3 (3-4)	4 (2-7)	6 (5-8)	
New CKD with diabetes; controlled BP, HbA_1c_, and FPG; not taking ACEI or ARB	0 (0-1)	2 (1-3)	4 (3-4)	0 (0-3)	3 (2-4)	
New CKD, no hypertension or diabetes	8 (7-8)	3 (2-4)	19 (17-20)	55 (46-64)	10 (8-13)	
Total	8 (8-9)	5 (4-7)	26 (24-27)	59 (50-69)	19 (16-23)	
NNS^d^	117 (107-130)	189 (149-259)	40 (38-42)	17 (15-20)	52 (44-62)	
**Sensitivity analysis 1**					
No CKD	970 (968-971)	971 (967-974)	888 (885-891)	852 (838-866)	919 (912-925)	
CKD but no change in treatment						
New CKD with hypertension^b^	1 (1-2)	1 (0-1)	6 (5-7)	18 (12-23)	13 (11-16)	
New CKD with diabetes^c^	1 (0-1)	1 (0-1)	8 (7-8)	12 (8-16)	9 (7-11)	
Known CKD	1 (1-2)	0 (0-1)	0 (0-1)	4 (2-7)	4 (3-5)	
Total	3 (2-3)	2 (1-2)	14 (13-15)	34 (26-41)	26 (22-30)	
Need for change in treatment without assessing eGFR or albuminuria						
New CKD with hypertension	10 (9-11)	7 (5-8)	35 (34-37)	24 (18-30)	16 (14-18)	
New CKD with diabetes	4 (4-5)	10 (8-12)	37 (36-39)	17 (12-22)	9 (8-11)	
Known CKD	3 (3-4)	1 (1-2)	0 (0-1)	4 (2-7)	7 (5-8)	
Total	17 (16-19)	18 (15-21)	72 (71-75)	45 (37-54)	32 (28-35)	
Need for change in treatment based on eGFR or albuminuria						
New CKD with hypertension, controlled BP, not taking ACEI or ARB	1 (0-1)	1 (0-2)	3 (3-4)	12 (7-16)	6 (5-8)	
New CKD with diabetes; controlled BP, HbA_1c_, and FPG; not taking ACEI or ARB	0 (0-1)	4 (3-6)	4 (3-4)	2 (1-4)	3 (3-5)	
New CKD, no hypertension or diabetes	9 (8-10)	4 (3-6)	19 (18-20)	55 (46-64)	14 (12-18)	
Total	10 (9-11)	9 (8-12)	26 (24-27)	69 (59-79)	23 (21-28)	
NNS^d^	102 (93-112)	101 (84-126)	39 (37-41)	14 (13-17)	41 (35-48)	
**Sensitivity analysis 2**					
No CKD	995 (994-995)	992 (990-993)	980 (979-981)	948 (940-957)	978 (975-981)	
CKD but no change in treatment						
New CKD with hypertension^b^	0 (0-1)	0 (0-1)	1 (1-2)	8 (4-11)	4 (3-5)	
New CKD with diabetes^c^	0 (0-1)	0 (0-1)	2 (1-2)	3 (1-6)	2 (2-3)	
Known CKD	1 (0-1)	0 (0-1)	0 (0-1)	3 (0-4)	2 (2-3)	
Total	1 (0-1)	0 (0-1)	3 (2-3)	14 (9-18)	8 (7-10)	
Need for change in treatment without assessing eGFR or albuminuria						
New CKD with hypertension	1 (1-2)	2 (1-3)	6 (5-7)	9 (5-12)	3 (2-5)	
New CKD with diabetes	1 (1-2)	3 (2-4)	8 (7-9)	7 (4-11)	3 (2-4)	
Known CKD	1 (1-2)	1 (0-1)	0 (0-1)	10 (6-14)	5 (3-6)	
Total	3 (3-4)	6 (4-7)	14 (13-15)	26 (20-32)	11 (9-12)	
Need for change in treatment based on eGFR or albuminuria						
New CKD with hypertension, controlled BP, not taking ACEI or ARB	0 (0-1)	0 (0-1)	0 (0-1)	0 (0-1)	1 (0-1)	
New CKD with diabetes; controlled BP, HbA_1c_, and FPG; not taking ACEI or ARB	0 (0-1)	1 (1-2)	1 (0-1)	1 (0-2)	1 (0-2)	
New CKD, no hypertension or diabetes	1 (0-1)	1 (0-2)	2 (2-3)	11 (7-15)	1 (0-1)	
Total	1 (1-2)	2 (2-4)	3 (3-4)	12 (8-17)	3 (2-4)	
NNS^d^	891 (702-1219)	351 (256-556)	324 (280-383)	81 (60-126)	323 (255-409)	

Abbreviations: ACEI, angiotensin-converting enzyme inhibitor; ARB, angiotensin receptor blocker; BP, blood pressure; CKD, chronic kidney disease; eGFR, estimated glomerular filtration rate; FPG, fasting plasma glucose; HbA_1c_, hemoglobin A_1c_; NNS, number needed to screen.

^a^ The definition of CKD was eGFR less than 60 mL/min/1.73 m^2^ in the primary definition, eGFR less than 60 mL/min/1.73 m^2^ or severe albuminuria in sensitivity analysis 1, and eGFR less than 45 mL/min/1.73 m^2^ in sensitivity analysis 2. Severe albuminuria was defined by any 1 of the following: an albumin-to-creatinine ratio of more than 300 mg/g or more than 30 mg/mmol, a protein-to-creatinine ratio of more than 500 mg/g or more than 50 mg/mmol, or a dipstick urinalysis result of 2 or greater for albumin.

^b^ New CKD with hypertension includes only individuals without diabetes. For participants with CKD but without diabetes, the need for a treatment change was defined by no use of ACEI or ARB or blood pressure greater than the target value of 140/90 mm Hg.

^c^ New CKD with diabetes includes individuals with or without hypertension. For participants with CKD and diabetes, the need for a treatment change was defined by no use of ACEI or ARB, blood pressure above the target value of 140/90 mm Hg, HbA_1c_ levels of 8% (0.08 of total hemoglobin) or more, or fasting glucose levels of 178.4 mg/dL (9.9 mmol/L) or more.

^d^ Results for the NNS as presented in the text cannot be directly calculated from the data in this table because of rounding.

### Sensitivity Analyses of Population-Based Screening

The prevalence of CKD using the first sensitivity definition (ie, eGFR < 60 mL/min/1.73 m^2^ or severe albuminuria) was 3.0% (95% CI, 2.9%-3.2%) in the China cohort, 2.9% (95% CI, 2.6%-3.3%) in the India cohort, 11.3% (95% CI, 11.0%-11.6%) in the Mexico cohort, 14.8% (95% CI, 13.4%-16.2%) in the Senegal cohort, and 8.1% (95% CI, 7.5%-8.8%) in the US cohort. Using the second sensitivity definition (ie, eGFR < 45 mL/min/1.73 m^2^ with or without severe albuminuria), the prevalence of CKD was 0.5% (95% CI, 0.5%-0.6%) for China, 0.8% (95% CI, 0.7%-1.0%) for India, 2.0% (95% CI, 1.9%-2.2%) for Mexico, 5.2% (95% CI, 4.3%-6.0%) for Senegal, and 2.2% (95% CI, 1.9%-2.5%) for the United States.

The proportion of participants for whom assessing CKD status as defined by eGFR less than 60 mL/min/1.73 m^2^ or severe albuminuria was associated with a change in treatment ranged from 9 individuals (95% CI, 8 individuals-12 individuals) per 1000 individuals for India to 69 individuals (95% CI, 59 individuals-79 individuals) per 1000 individuals for Senegal (Table 2; eFigures 1 and 2 in the Supplement). Using this definition of CKD, the NNS to find 1 participant in whom assessing CKD status was associated with a change in treatment was 102 individuals (95% CI, 93 individuals-112 individuals) for China, 101 individuals (95% CI, 84 individuals-126 individuals) for India, 39 individuals (95% CI, 37 individuals-41 individuals) for Mexico, 14 individuals (95% CI, 13 individuals-17 individuals) for Senegal, and 41 individuals (95% CI, 35 individuals-48 individuals) for the United States.

The proportion of participants for whom assessing CKD status as defined by eGFR less than 45 mL/min/1.73 m^2^ was associated with a change in treatment ranged from 2 individuals (95% CI, 2 individuals-4 individuals) per 1000 individuals for China to 12 individuals (95% CI, 8 individauls-17 individuals) per 1000 individuals for Senegal (Table 2; eFigures 3 and 4 in the Supplement). When CKD was defined by eGFR less than 45 mL/min/1.73 m^2^, the NNS to find 1 participant for whom assessing CKD status was associated with a change in treatment was 891 individuals (95% CI, 702 individuals-1219 individuals) for China, 351 individuals (95% CI, 256 individuals-556 individuals) for India, 324 individuals (95% CI, 280 individuals-383 individuals) for Mexico, 81 individuals (95% CI, 60 individuals-126 individuals) for Senegal, and 323 individuals (95% CI, 255 individuals-409 individuals) for the United States. Findings were not sensitive to varying the proportion of ACEI or ARB use in China or Mexico (eTables 1, 2, and 3 in the Supplement).

### Yield of Case Finding

CKD status was assessed among adults with a self-reported history of hypertension, diabetes, or CKD and those with blood pressure levels of 140/90 mm Hg or more or laboratory evidence of diabetes, HbA_1c_ levels of 6.5% (0.065 of total hemoglobin) or more, or fasting blood glucose levels of 126.1 mg/dL or more. When CKD was assessed in this subroup (case finding), the number of participants who required assessment of CKD status was decreased while the proportion of participants with CKD who required a treatment change was similar or was increased compared with screening the entire population (Table 3). Specifically, the number of individuals requiring assessment of CKD status decreased by as little as 38.4% (95% CI, 38.0%-38.8%) for Mexico and as much as 59.3% (95% CI, 58.8%-59.7%) for China, whereas the proportion of individuals with CKD who required a treatment change increased by as much as 89.6% (95% CI, 80.4%-99.3%) for the United States. Case finding was also associated with a decrease in the proportion of individuals needing GFR measurements of as much as 57.8% (95% CI, 56.3%-59.3%) in the US. The proportion of all individuals with CKD detected by case finding ranged from 46.2% (95% CI, 45.1%-47.4%) for Senegal to 86.4% (95% CI, 85.4%-87.3%) for India; the median proportion was 79.8% (95% CI, 79.8%-79.9%).

**Table 3.** Testing Requirements and Yield of Screening vs Case Finding

Strategy^a^	% (95% CI)^b^				
	China	India	Mexico	Senegal	United States
Screening^c^					
Measuring eGFR required, No.	47 204	9817	51 137	2441	223M
Individuals identified with CKD requiring treatment change, No.	1065	220	4701	251	10.4 million
Proportion	2.3 (2.1-2.4)	2.2 (1.9-2.5)	9.2 (8.9-9.4)	10.3 (9.1-11.5)	4.7 (4.2-5.2)
Change required based on eGFR measurement, No.	403	52	1286	145	4.3 million
Proportion	0.9 (0.8-0.9)	0.5 (0.4-0.7)	2.5 (2.4-2.7)	5.9 (5.0-6.9)	1.9 (1.6-2.3)
Case finding^d^					
Measuring eGFR required, No.	19 234	5348	31 489	1106	93.9 million
Individuals identified with CKD requiring treatment change, No.	704	190	3753	116	8.4 million
Proportion	3.7 (3.4-3.9)	3.6 (3.1-4.1)	11.9 (11.5-12.3)	10.5 (8.7-12.3)	8.9 (8.1-9.8)
Implications of case finding vs screening					
Decrease in proportion of individuals with recommended eGFR measurement	59.3 (58.8-59.7)	45.5 (44.5-46.5)	38.4 (38.0-38.8)	54.7 (52.7-56.7)	57.8 (56.3-59.3)
Increase in proportion of individuals with detected CKD requiring treatment change	62.2 (59.3-65.1)	58.5 (52.0-65.0)	29.6 (28.3-31.0)	2.0 (0.3-3.7)	89.6 (80.4-99.3)
Proportion of individuals with CKD identified with case-finding strategy	66.1 (65.9-66.3)	86.4 (85.4-87.3)	79.8 (79.8-79.9)	46.2 (45.1-47.4)	86.3 (83.1-89.0)
Proportion of individuals with treatment change requiring eGFR to be detected	37.8 (37.5-38.1)	23.6 (21.8-25.5)	27.4 (27.3-27.4)	57.8 (56.7-58.8)	42.9 (38.5-47.4)

Abbreviations: CKD, chronic kidney disease; eGFR, estimated glomerular filtration rate; HbA_1c_, hemoglobin A_1c_.

^a^ CKD was defined using the primary definition (ie, eGFR < 60 mL/min/1.73 m^2^).

^b^ Results as presented in the text cannot be directly calculated from the data in this table because of rounding.

^c^ Screening was defined by measuring eGFR in all adults from the target population.

^d^ Case finding was defined by measuring eGFR only in adults with a history of hypertension, diabetes, or CKD; with blood pressure levels of 140/90 mm Hg or more; or with laboratory evidence of diabetes (ie, HbA_1c_ ≥ 6.5 [0.065 of total hemoglobin] or fasting blood glucose > 126 mg/dL [7.0 mmol/L]).

## Discussion

In this epidemiologic assessment of population-based cohorts from China, India, Mexico, Senegal, and the United States, the prevalence of CKD as defined by eGFR less than 60 mL/min/1.73 m^2^ ranged from 2.3% to 13.1%. In all 5 settings, fewer than 15% of participants with CKD were aware that they had this condition. Among those with CKD, treatment gaps were relatively common, ranging from 68.7% to 97.8%. These findings may suggest that screening for CKD is associated with a clinical benefit. However, if renin-angiotensin system inhibitors were used as first-line agents among individuals with uncontrolled blood pressure, simply measuring blood pressure was associated with identification of most individuals for whom a treatment change was required; measuring an index of glycemic control was associated with a further increase in this yield. Measuring eGFR or albuminuria was not associated with frequent identification of an indication for a treatment change, suggesting that CKD screening programs may not be associated with a benefit for most participants.

Within the populations studied, those in Senegal and Mexico had more favorable NNS values (17 and 40, respectively), whereas India had a less favorable NNS (189) and the United States and China had intermediate NNS values (52 and 117, respectively). These results suggest that although the wealth of a country may be associated with the yield of screening for CKD, other factors, such as the prevalence of CKD and other NCDs, access to health care, and the availability of low-cost NCD treatment, may also be associated with this outcome. In addition, some regions may have an increased prevalence of CKD without accompanying diabetes or uncontrolled blood pressure; this nontraditional CKD^28,29^ has been associated with infections, environmental and occupational exposures, and kidney stones. Additionally, because agricultural populations in certain areas have an increased incidence of deaths attributed to kidney disease^30,31^ and because rural areas may have decreased access to specialist care, the yield of population-based CKD screening may differ by urban vs rural status.

Because it is more economically efficient to treat individuals with known conditions rather than searching for new diagnoses, it may appear more rational to allocate resources to increasing drug coverage for individuals with previously identified NCDs rather than to population-based screening. However, the decision to screen may appropriately consider other factors, such as raising awareness, mapping local causes of CKD, and responding to perceived population burden.

If early detection of CKD is desired, our findings suggest that case finding is more efficient and cost-effective than population-based screening. Case finding was associated with a significant decrease in the number of individuals who required eGFR testing and increase in the proportion of individuals with CKD for whom a treatment change was indicated. This builds on previous work suggesting that case finding is associated with favorable cost utility.^32^ Case finding was associated with the identification of most individuals with CKD for 4 of 5 countries studied but would miss 53.8% of individuals with CKD in the Senegal cohort, perhaps associated with decreased access to specialist care in this rural population. These results suggest that further work is needed to assess the optimal strategy for case finding and determine how best to integrate case finding for CKD with treatment of other NCDs. As for all early detection programs, jurisdictions that pursue case finding should attempt to minimize overtesting and harms associated with testing.

Prior work has focused on the proportion of individuals who are identified as having CKD, assuming that treatment would change for most or all such individuals.^33^ Our results suggest that although population-based screening for CKD may be associated with identification of a large number of individuals whose treatment should change, the need for these changes would be similarly apparent after simply assessing blood pressure, assessing glycemic control, and using an ACEI or ARB as first-line therapy for individuals with hypertension. There may be other benefits associated with early detection of CKD, such as increased use of living donor transplantation, more time to discuss an appropriate modality choice for those who require kidney replacement, more cautious use of intravenous contrast, up-titration of existing ACEI or ARB regimens, discontinuation of nephrotoxic medications, or the opportunity to adjust drug dosing for eGFR.^33^ In addition, multiple measurements of eGFR even when the initial value is more than 60 ml/min/1.73 m^2^ may help to trigger early intervention for individuals with rapid loss of kidney function. However, these speculative benefits seem unlikely to be associated with large differences in health outcomes on a population level,^16^ and a strategy based on multiple measurements among individuals would be very resource intensive.

We defined adequate blood pressure control as levels less than 140/90 mm Hg. The 2017 American College of Cardiology/American Heart Association guideline recommends a blood pressure target of less than 130/80 mm Hg for almost all adults, including those with CKD.^26^ If blood pressure control were defined as in this guideline, a smaller number of individuals would have controlled BP and the potential benefit associated with screening for CKD would be smaller than we estimated in this study. Conversely, the 2021 Kidney Disease Improving Global Outcomes guideline recommends control of systolic blood pressure to less than 120 for most adults with CKD.^34^ Using a target of less than 130/80 mm Hg for individuals without CKD and a target of less than 120 for individuals with CKD may be associated with increased numbers of patients for whom screening or case finding with eGFR and albuminuria would be associated with a treatment change. This is because as many as 20% of US adults with CKD may have systolic blood pressure from 120 to 130.^35^

### Limitations

There were some differences in the design of the cohorts (eg, the Mexico study excluded individuals who reported that they had CKD, whereas the other studies did not) and in the demographic characteristics of participants across cohorts, likely associated with differences in the source populations. The consistency of our conclusions across these different settings may increase confidence in the findings. However, our analysis also has several limitations that should be considered. First, 3 of 5 cohorts were subnational samples rather than nationally representative samples. However, the consistency of the results across all 5 settings suggests that it is unlikely that exclusive use of national data would have influenced our conclusions. Second, we did not have data on family history of CKD or occupation, which could also be used to inform case finding. Third, we defined CKD based on a single measurement of kidney function. Although this may have been associated with an overestimation of CKD prevalence, it should not have affected our conclusions because obtaining a confirmatory measurement would be associated with decreased prevalence of screen-detected CKD. Fourth, we estimated the proportion of participants in China and Mexico who were using ACEI or ARB using data from the United States. However, a sensitivity analysis suggests that this assumption was associated with little change in the study results. Fifth, we could have considered the use of statins and SGLT2 inhibitors, which are associated with improved outcomes among individuals with CKD. However, the high cost of SGLT2 inhibitors suggests that they will be infrequently used in LMICs at present and reinforces the opportunity cost associated with detecting additional cases of CKD as opposed to treating known cases. Sixth, our findings may not apply in settings where ACEI and ARB are infrequently used as first-line treatment for hypertension among individuals without CKD. Seventh, other system-level characteristics, such as health insurance coverage, accessibility of health services, and national priorities, may make early detection more or less appealing.

## Conclusions

These findings suggest that measuring eGFR or albuminuria in population-based screening programs may not be associated with more frequent identification of an indication for a change in treatment in comparison with simply measuring blood pressure, inquiring about antihypertensive medication use, assessing glycemic control, and first-line use of ACEI or ARB therapy among individuals with diabetes or hypertension. These data add to evidence suggesting that population-based CKD screening is not a wise use of resources but may warrant re-evaluation if SGLT2 inhibitors become less expensive and more widely available, especially in LMICs. If the early detection of CKD is desired, case finding may be appropriate, given that it was associated with an increase in the yield of individuals who required a treatment change and a decrease in the need for diagnostic testing.

## Notes

Supplement. **eMethods.** Study Cohort Descriptions **eFigure 1.** Yield of Screening for Chronic Kidney Disease in Sensitivity Analysis 1 **eFigure 2.** Indications for Changes in Treatment in Screen-detected Diagnoses by Presence or Absence of Hypertension, Diabetes, and Chronic Kidney Disease in Sensitivity Analysis 1 **eFigure 3.** Yield of Screening for Chronic Kidney Disease in Sensitivity Analysis 2 **eFigure 4.** Indications for Changes in Treatment in Screen-detected Diagnoses by Presence or Absence of Hypertension, Diabetes, and Chronic Kidney Disease in Sensitivity Analysis 2 **eTable 1.** Proportion of Individuals With Treatment Change Needed in China and Mexico When Drug Use Varied vs the US **eTable 2.** Yield of Screening per 1000 Individuals Screened in China and Mexico When Drug Use Varied vs the US **eTable 3.** Testing Requirements and Yield of Screening vs Case Finding Using Primary Chronic Kidney Disease Definition in China and Mexico When Drug Use Varied vs the US
eReferences.
