# Supplementary material for: Diagnostic Yield of Population-Based Screening for Chronic Kidney Disease in Low-Income, Middle-Income, and High-Income Countries
Source: JAMA Netw Open. 2021 Oct 4;4(10):e2127396. doi: 10.1001/jamanetworkopen.2021.27396 (PMC8491102; doi:10.1001/jamanetworkopen.2021.27396)
Supplement: Supplement. — eMethods. Study Cohort Descriptions eFigure 1. Yield of Screening for Chronic Kidney Disease in Sensitivity Analysis 1 eFigure 2. Indications for Changes in Treatment in Screen-detected Diagnoses by Presence or Absence of Hypertension, Diabetes, and Chronic Kidney Disease in Sensitivity Analysis 1 eFigure 3. Yield of Screening for Chronic Kidney Disease in Sensitivity Analysis 2 eFigure 4. Indications for Changes in Treatment in Screen-detected Diagnoses by Presence or Absence of Hypertension, Diabetes, and Chronic Kidney Disease in Sensitivity Analysis 2 eTable 1. Proportion of Individuals With Treatment Change Needed in China and Mexico When Drug Use Varied vs the US eTable 2. Yield of Screening per 1000 Individuals Screened in China and Mexico When Drug Use Varied vs the US eTable 3. Testing Requirements and Yield of Screening vs Case Finding Using Primary Chronic Kidney Disease Definition in China and Mexico When Drug Use Varied vs the US eReferences. [file jamanetwopen-e2127396-s001.pdf]

Supplemental Online Content

Tonelli M, Tiv S, Anand S, et al. Diagnostic yield of population-based screening for chronic kidney disease in low-income, middle-income, and high-income countries. *JAMA Netw Open*. 2021;4(10):e2127396. doi:10.1001/jamanetworkopen.2021.27396

eMethods. Study Cohort Descriptions

eFigure 1. Yield of Screening for Chronic Kidney Disease in Sensitivity Analysis 1

eFigure 2. Indications for Changes in Treatment in Screen-detected Diagnoses by Presence or Absence of Hypertension, Diabetes, and Chronic Kidney Disease in Sensitivity Analysis 1

eFigure 3. Yield of Screening for Chronic Kidney Disease in Sensitivity Analysis 2

eFigure 4. Indications for Changes in Treatment in Screen-detected Diagnoses by Presence or Absence of Hypertension, Diabetes, and Chronic Kidney Disease in Sensitivity Analysis 2

eTable 1. Proportion of Individuals With Treatment Change Needed in China and Mexico When Drug Use Varied vs the US

eTable 2. Yield of Screening per 1000 Individuals Screened in China and Mexico When Drug Use Varied vs the US

eTable 3. Testing Requirements and Yield of Screening vs Case Finding Using Primary Chronic Kidney Disease Definition in China and Mexico When Drug Use Varied vs the US

eReferences.

This supplemental material has been provided by the authors to give readers additional information about their work.

© 2021 Tonelli M et al. *JAMA Network Open.*

eMethods. Study Cohort Descriptions

China
The China cohort was based on a representative sample of people aged 18 years or older in 13 Chinese provinces identified using a stratified, multistage probability sampling design. Data were collected in examination centres at local health stations or community clinics in the participants' residential area. Questionnaires were used to obtain information on age, sex, education, income, smoking status, history of diabetes, hypertension and kidney disease and medication use. Blood pressure, height and weight were measured and samples of blood and urine were collected. Blood pressure was measured by sphygmomanometer, three times at 1-minute intervals. The mean of the three readings was calculated, unless the difference between the readings was greater than 10 mm Hg, in which case the mean of the two closest measurements was used. Fasting glucose and creatinine were measured using blood collected at the study visit; estimated glomerular filtration rate (eGFR) was calculated using serum creatinine and an estimating equation derived for use in Chinese chronic kidney disease (CKD) patients. Urinary albumin and creatinine were measured from a fresh morning spot urine sample or morning urine sample stored at 4°C for less than 1 week and albumin-to-creatinine ratio (ACR) was calculated.

India
The India cohort was based on a representative sample of people aged 20 years or older in 2 Indian cities (Chennai, Delhi) identified using a multistage clustered probability sampling design. Data collected during study visits to the participants' homes included age, sex, education, income, smoking status, history of diabetes, hypertension and kidney disease and medication use. Blood pressure, height and weight were measured and samples of blood and urine were collected. Blood pressure was measured by electronic sphygmomanometer according to published guidelines.(1) Fasting glucose and creatinine were measured using blood collected at the study visit; estimated glomerular filtration rate (eGFR) was calculated using serum creatinine and the Chronic Kidney Disease Epidemiology Collaboration (CKD-EPI) estimating equation. Urinary albumin and creatinine were measured from morning spot urine samples that were stored at -80 degrees until the assays were done, and ACR was calculated.

Mexico
The Mexico cohort was based on participants in a community-based program that evaluated adult residents of the Jalisco state for CKD and other noncommunicable diseases (NCD). Individuals who reported being aware that they had kidney disease were excluded from participation. Data were collected by mobile units that traveled around the state, and which typically remained at each site for 5 consecutive days. Data were collected using a standardized form, including age, sex, education, smoking status, alcohol use, diabetic status, and personal history of diabetes or hypertension. Blood pressure, height and weight were measured, and samples of blood and urine were collected. Systolic and diastolic blood pressure were measured by trained personnel using manual sphygmomanometers after participants had rested quietly for 5 minutes. Fasting blood glucose and serum creatinine were measured and glomerular filtration rate (GFR) was estimated with the Chronic Kidney Disease Epidemiology Collaboration (CKD-EPI) equation.(2) Dipstick urinalysis was performed and interpreted by trained experienced personnel working in well-lit and appropriate working conditions.

Senegal
The Senegal cohort was based on a community-based cross-sectional survey of a sample drawn from individuals aged ≥18 years and living in Saint-Louis, Senegal for at least 3 months. A two-stage cluster sampling method was used to select a representative sample of adults living in urban and rural areas of Saint-Louis. Data were collected during home visits or at the nearest health centre. Questionnaires were used to obtain information on age, race, sex, education, income, smoking status, history of diabetes and hypertension, and medication use. Blood pressure, height and weight were measured and samples of blood and urine were collected. Blood pressure was measured twice at five-minute intervals using a semiautomatic sphygmomanometer and the mean of the two readings was calculated. If the difference between the readings was greater than 10 mm Hg, a third measurement was performed. Fasting blood glucose was measured using a glucose oxidase method. Serum creatinine was measured and glomerular filtration rate (GFR) was estimated with the Modification of Diet in Renal Disease (MDRD) equation. Urine samples were first tested using dipsticks and those with >2+ albuminuria had urine albumin quantified in a second 24h urine sample.

United States

The United States cohort included the National Health and Nutrition Examination Survey (NHANES), which was based on three samples of the non-institutionalized civilian United States population aged ≥ 20 years of age identified using a stratified, multistage probability sampling approach in 2009-2010, 2011-2012, and 2013-2014. Questionnaires were used to obtain information on age, race/ethnicity, sex, education, income, smoking status, history of diabetes and hypertension, and antihypertensive and glucose-lowering medication use. The use of ACEI/ARB was determined through a medication inventory. Blood pressure, height and weight were measured and blood and random spot urine samples were collected. Blood pressure was measured three times following a standardized protocol and averaged for all analyses. Serum glucose, glycated hemoglobin, and creatinine were measured using blood collected at the study visit; estimated glomerular filtration rate (eGFR) was calculated using serum creatinine and the CKD-EPI equation.(2) Urinary albumin and creatinine were measured from a spot urine specimen and ACR was calculated. Data for NHANES were weighted to represent the non-institutionalized civilian United States population and accounted for the complex survey design used in selecting participants.

© 2021 Tonelli M et al. *JAMA Network Open.*

eFigure 1. Yield of Screening for Chronic Kidney Disease in Sensitivity Analysis 1

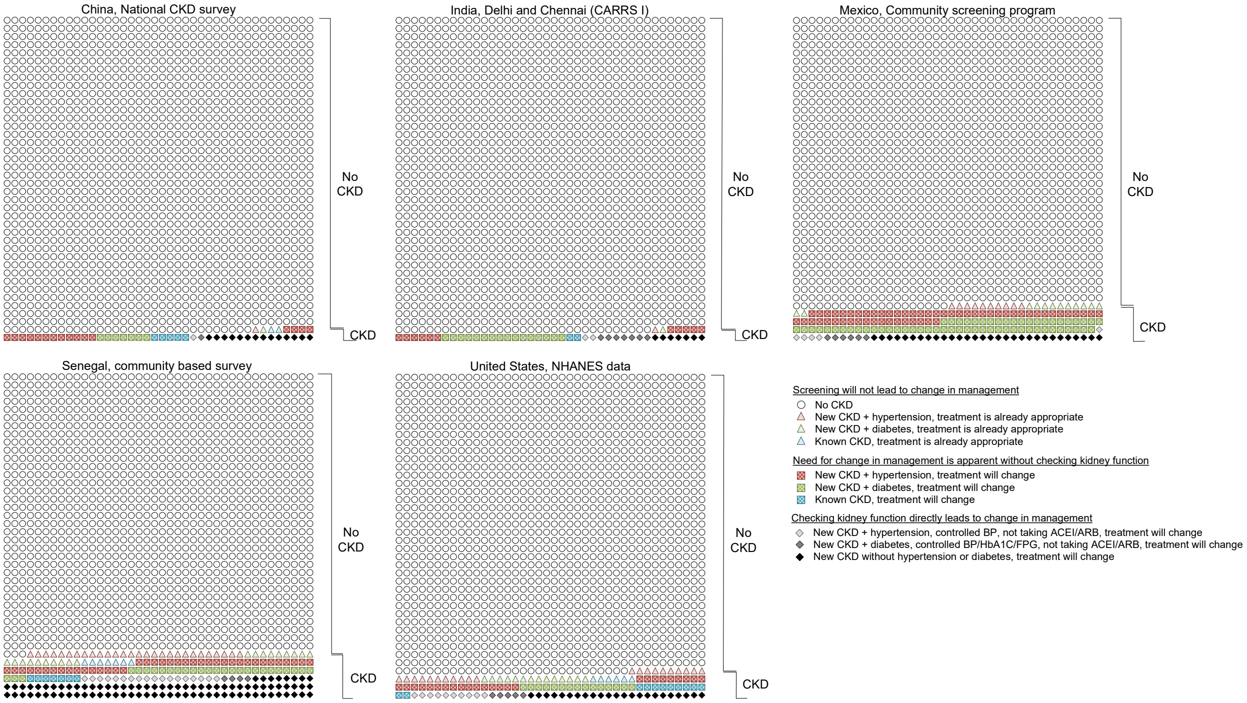

CKD was defined by eGFR<60 ml/min/1.73m^2 or severe albuminuria. The Figure graphically presents the number of cases in each category, expressed per 1600 people. Data were based on Table 2. New CKD + hypertension includes only people without diabetes. New CKD + diabetes includes people with or without hypertension.

CKD – chronic kidney disease, eGFR – estimated glomerular filtration rate, CARRS – Centre for Cardiometabolic Risk Reduction in South-Asia, NHANES – National Health and Nutrition Examination Survey, BP – blood pressure, ACEI/ARB – angiotensin converting enzyme inhibitor or angiotensin receptor blocker, HbA1C – hemoglobin A1C, FPG – fasting plasma glucose

© 2021 Tonelli M et al. *JAMA Network Open.*

eFigure 2. Indications for Changes in Treatment in Screen-detected Diagnoses by Presence or Absence of Hypertension, Diabetes, and Chronic Kidney Disease in Sensitivity Analysis 1

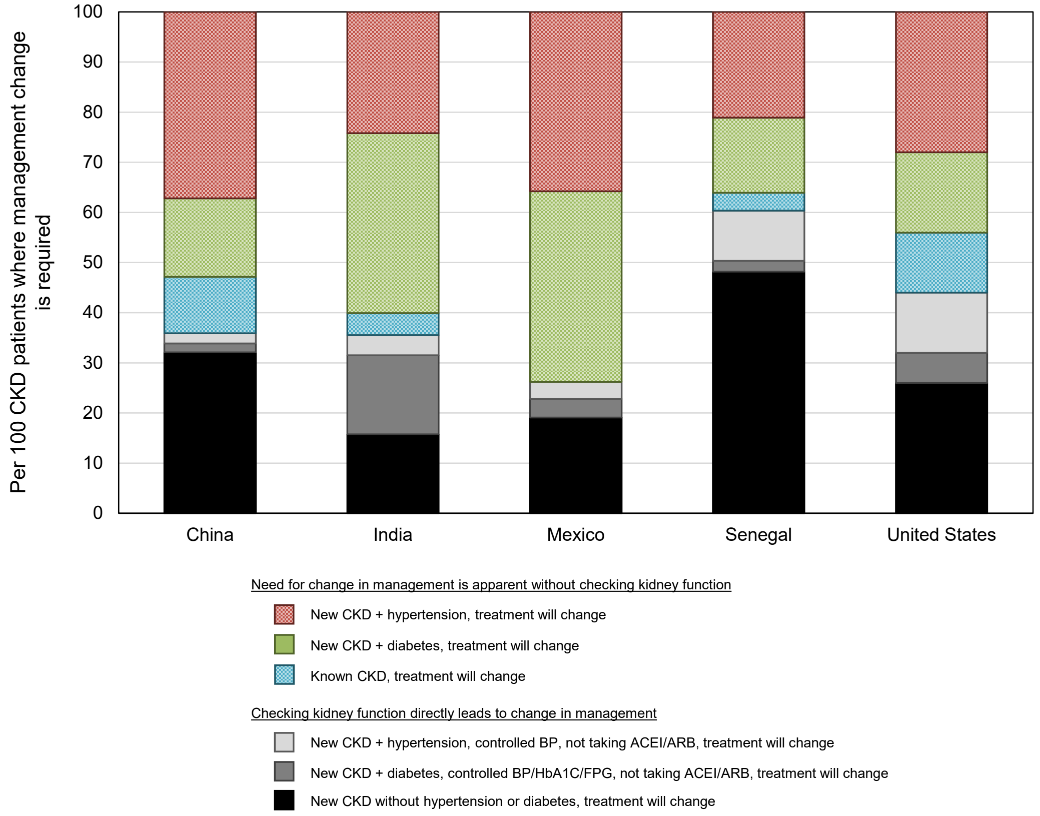

CKD was defined by eGFR<60 ml/min/1.73m^2 or severe albuminuria. New CKD + hypertension includes only people without diabetes. New CKD + diabetes includes people with or without hypertension.

CKD – chronic kidney disease, eGFR – estimated glomerular filtration rate, BP – blood pressure, ACEI/ARB – angiotensin converting enzyme inhibitor or angiotensin receptor blocker, HbA1C – hemoglobin A1C, FPG – fasting plasma glucose

© 2021 Tonelli M et al. *JAMA Network Open.*

eFigure 3. Yield of Screening for Chronic Kideny Disease in Sensitivity Analysis 2

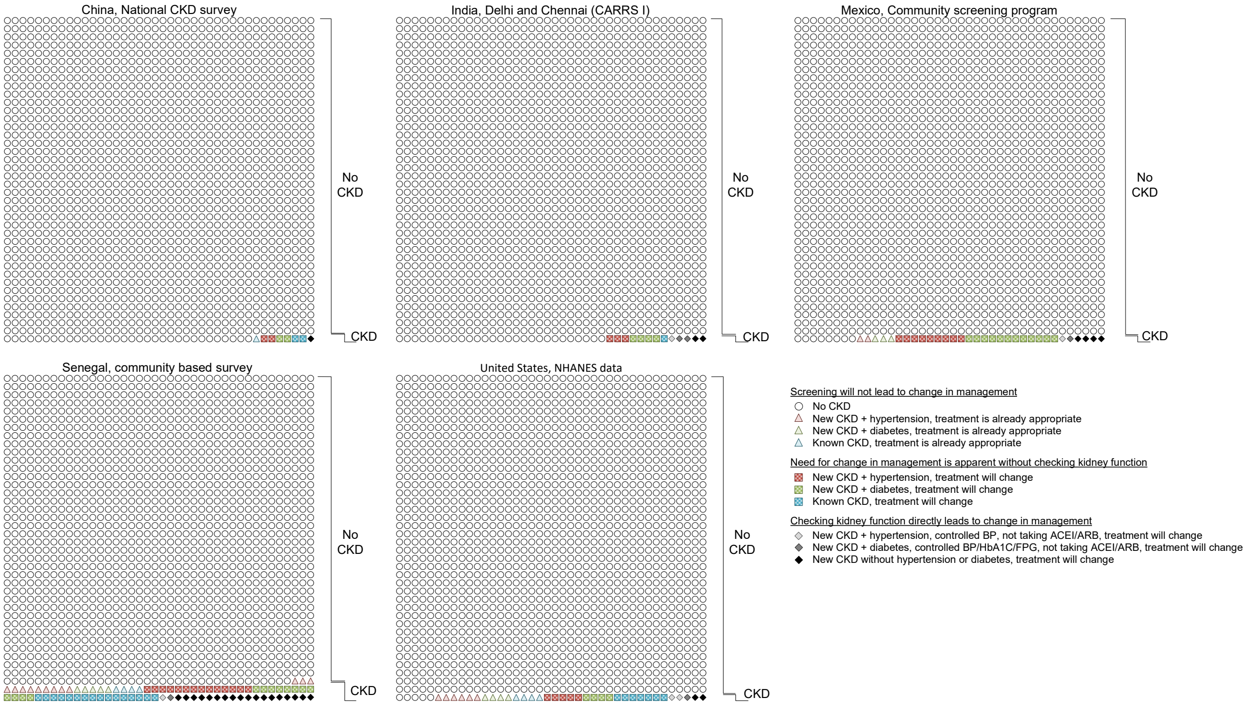

CKD was defined by eGFR<45 ml/min/1.73m^2 with or without severe albuminuria. The Figure graphically presents the number of cases in each category. Data were based on Table 2. New CKD + hypertension includes only people without diabetes. New CKD + diabetes includes people with or without hypertension.

CKD – chronic kidney disease, eGFR – estimated glomerular filtration rate, CARRS – Centre for Cardiometabolic Risk Reduction in South-Asia, NHANES – National Health and Nutrition Examination Survey, BP – blood pressure, ACEI/ARB – angiotensin converting enzyme inhibitor or angiotensin receptor blocker, HbA1C – hemoglobin A1C, FPG – fasting plasma glucose

© 2021 Tonelli M et al. *JAMA Network Open.*

eFigure 4. Indications for Changes in Treatment in Screen-detected Diagnoses by Presence or Absence of Hypertension, Diabetes, and Chronic Kidney Disease in Sensitivity Analysis 2

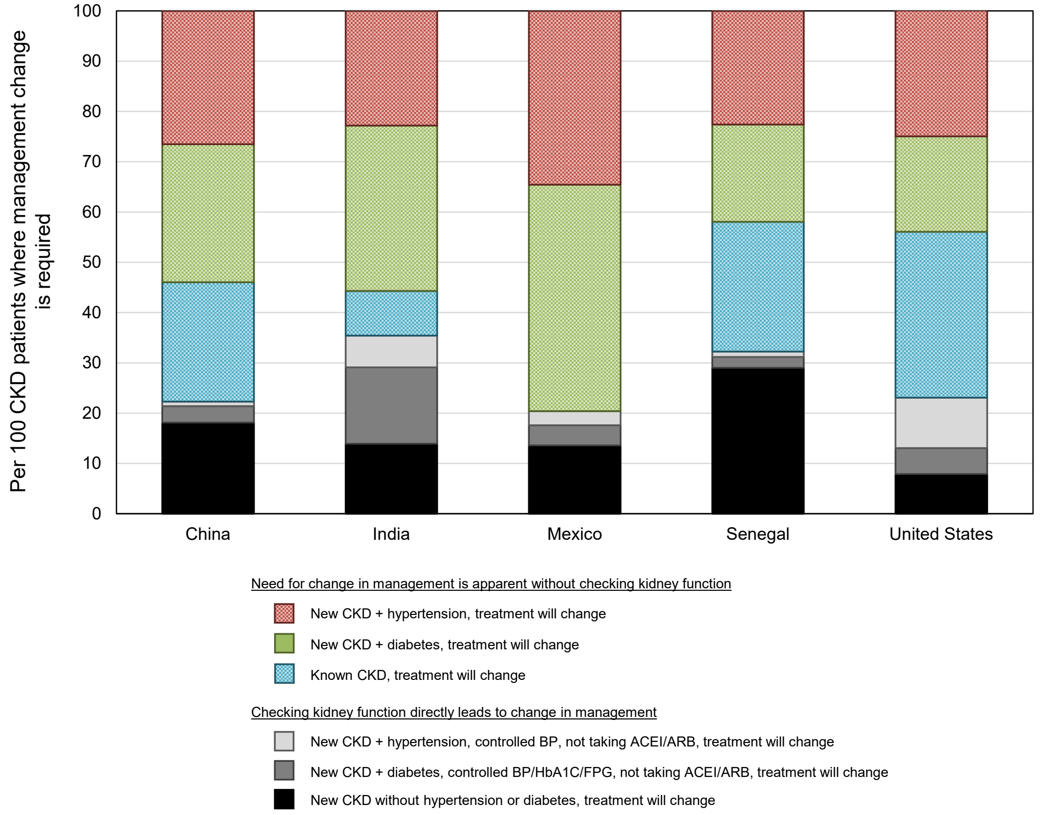

CKD was defined by eGFR<45 ml/min/1.73m^2. New CKD + hypertension includes only people without diabetes. New CKD + diabetes includes people with or without hypertension.

CKD – chronic kidney disease, eGFR – estimated glomerular filtration rate, BP – blood pressure, ACEI/ARB – angiotensin converting enzyme inhibitor or angiotensin receptor blocker, HbA1C – hemoglobin A1C, FPG – fasting plasma glucose

© 2021 Tonelli M et al. *JAMA Network Open.*

eTable 1. Proportion of Individuals With Treatment Change Needed in China and Mexico When Drug Use Varied vs the US

Characteristic	China			Mexico		
	Using ACEI/ARB estimate from US	10% higher than US	10% lower than US	Using ACEI/ARB estimate from US	10% higher than US	10% lower than US
Primary definition of CKD: eGFR<60 ml/min/1.73m^2						
N with CKD	1,185	1,185	1,185	5,413	5,413	5,413
N with CKD needing management change	1,065	1,073	1,058	4,701	4,735	4,667
Proportion with CKD in whom a management change is needed	89.9 (88.2-91.6)	90.6 (88.9-92.2)	89.3 (87.5-91)	86.8 (85.9-87.7)	87.5 (86.6-88.3)	86.2 (85.3-87.1)
Sensitivity analysis 1: eGFR<60 ml/min/1.73m^2 or severe albuminuria						
N with CKD	1,427	1,427	1,427	5,779	5,779	5,779
N with CKD needing management change	1,293	1,303	1,285	5,038	5,074	5,002
Proportion with CKD in whom a management change is needed	90.6 (89.1-92.1)	91.3 (89.8-92.8)	90.0 (88.5-91.6)	87.2 (86.3-88.0)	87.8 (87.0-88.6)	86.6 (85.7-87.4)
Sensitivity analysis 2: eGFR<45 ml/min/1.73m^2						
N with CKD	245	245	245	1,043	1,043	1,043
N with CKD needing management change	215	217	213	891	897	885
Proportion with CKD in whom a management change is needed	87.8 (83.7-91.9)	88.5 (84.5-92.5)	86.9 (82.7-91.1)	85.4 (83.3-87.6)	86.0 (83.9-88.1)	84.9 (82.7-87.0)

Proportions in the table are presented as percentage (95% confidence interval)

CKD – chronic kidney disease, ACEI/ARB – angiotensin converting enzyme inhibitor or angiotensin receptor blocker, US – United States.

© 2021 Tonelli M et al. *JAMA Network Open.*

eTable 2. Yield of Screening per 1000 Individuals Screened in China and Mexico When Drug Use Varied vs the US

Group	China			Mexico		
	Using ACE/ARB estimate from US	10% higher than US	10% lower than US	Using ACE/ARB estimate from US	10% higher than US	10% lower than US
**Primary Definition: CKD defined by eGFR<60 ml/min/1.73m^2**						
**No CKD**						
No CKD	975 (973-976)	975 (973-976)	975 (973-976)	895 (892-897)	895 (892-897)	895 (891-897)
**CKD, but no change in management**						
New CKD + hypertension, management appropriate	1 (1-2)	1 (1-2)	1 (1-2)	6 (5-7)	6 (5-6)	6 (6-7)
New CKD + diabetes, management appropriate	0 (0-1)	0 (0-1)	1 (0-1)	7 (7-8)	7 (6-8)	7 (7-9)
Known CKD, management appropriate	1 (1-2)	1 (1-2)	1 (1-2)	0 (0-1)	0 (0-1)	0 (0-1)
Total CKD, but no change in management	2 (2-3)	2 (2-3)	3 (2-3)	13 (12-14)	13 (12-14)	13 (13-15)
**Need for change in management is apparent without assessing eGFR or albuminuria**						
New CKD + hypertension, management will change	9 (8-9)	9 (8-9)	8 (8-9)	34 (33-36)	34 (33-36)	34 (33-36)
New CKD + diabetes, management will change	3 (2-3)	3 (2-3)	3 (2-3)	32 (31-34)	32 (31-34)	32 (31-34)
Known CKD, management will change	3 (2-3)	3 (2-3)	3 (2-3)	0 (0-1)	0 (0-1)	0 (0-1)
Total need for change in management is apparent without assessing eGFR or albuminuria	15 (13-15)	15 (13-15)	14 (13-15)	66 (65-69)	66 (65-69)	66 (65-69)
**Need for change in management based on assessing eGFR and/or albuminuria directly**						
New CKD + hypertension, controlled BP, not taking ACEI/ARB, management will change	0 (0-1)	0 (0-1)	0 (0-1)	3 (3-4)	3 (3-4)	3 (2-3)
New CKD + diabetes, controlled BP/HbA1C/FPG, not taking ACEI/ARB, management will change	0 (0-1)	0 (0-1)	0 (0-1)	4 (3-4)	4 (3-4)	4 (3-4)

© 2021 Tonelli M et al. *JAMA Network Open.*

Group	China			Mexico		
	Using ACE/ARB estimate from US	10% higher than US	10% lower than US	Using ACE/ARB estimate from US	10% higher than US	10% lower than US
New CKD, no hypertension or diabetes, management will change	8 (7-8)	8 (7-8)	8 (7-8)	19 (17-20)	19 (17-20)	19 (17-20)
Total need for change in management based on assessing eGFR and/or albuminuria directly	8 (8-9)	8 (8-9)	8 (8-9)	26 (24-27)	26 (24-27)	26 (23-26)
Number needed to screen	117 (107-130)	116 (106-128)	118 (108-131)	40 (38-42)	39 (37-41)	41 (39-43)
**Sensitivity Analysis 1: CKD defined by (eGFR<60 ml/min/1.73m^2 or severe albuminuria)**						
**No CKD**						
No CKD	970 (968-971)	970 (968-971)	970 (968-971)	888 (885-891)	888 (885-891)	888 (885-891)
**CKD, but no change in management**						
New CKD + hypertension, management appropriate	1 (1-2)	1 (1-2)	1 (1-2)	6 (5-7)	6 (5-6)	6 (6-7)
New CKD + diabetes, management appropriate	1 (0-1)	1 (0-1)	1 (0-1)	8 (7-8)	7 (7-8)	8 (7-9)
Known CKD, management appropriate	1 (1-2)	1 (1-2)	1 (1-2)	0 (0-1)	0 (0-1)	0 (0-1)
Total CKD, but no change in management	3 (3-4)	3 (2-3)	3 (3-4)	14 (13-15)	13 (12-14)	14 (13-15)
**Need for change in management is apparent without assessing eGFR or albuminuria**						
New CKD + hypertension, management will change	10 (9-11)	10 (9-11)	10 (9-11)	35 (34-37)	35 (34-37)	35 (34-37)
New CKD + diabetes, management will change	4 (4-5)	4 (4-5)	4 (4-5)	37 (36-39)	37 (36-39)	38 (36-39)

© 2021 Tonelli M et al. *JAMA Network Open.*

Group	China			Mexico		
	Using ACE/ARB estimate from US	10% higher than US	10% lower than US	Using ACE/ARB estimate from US	10% higher than US	10% lower than US
Known CKD, management will change	3 (3-4)	3 (3-4)	3 (3-4)	0 (0-1)	0 (0-1)	0 (0-1)
Total need for change in management is apparent without assessing eGFR or albuminuria	17 (16-19)	17 (16-19)	17 (16-19)	72 (71-75)	72 (71-75)	73 (71-75)
**Need for change in management based on assessing eGFR and/or albuminuria directly**						
New CKD + hypertension, controlled BP, not taking ACEI/ARB, management will change	1 (0-1)	1 (0-1)	1 (0-1)	3 (3-4)	4 (3-4)	3 (3-4)
New CKD + diabetes, controlled BP/HbA1C/FPG, not taking ACEI/ARB, management will change	0 (0-1)	0 (0-1)	0 (0-1)	4 (3-4)	4 (4-5)	3 (3-4)
New CKD, no hypertension or diabetes, management will change	9 (8-10)	9 (8-10)	9 (8-10)	19 (18-20)	19 (18-20)	19 (17-20)
Total need for change in management based on assessing eGFR and/or albuminuria directly	10 (9-11)	10 (9-11)	10 (9-11)	26 (24-27)	27 (25-28)	25 (24-26)
Number needed to screen	102 (93-112)	101 (92-110)	103 (94-113)	39 (37-41)	38 (36-40)	40 (38-43)
**Sensitivity Analysis 2: CKD defined by eGFR<45 ml/min/1.73m^2 with or without albuminuria**						
**No CKD**						
No CKD	995 (994-995)	995 (994-995)	995 (994-995)	980 (979-981)	980 (979-981)	980 (979-981)
**CKD, but no change in management**						
New CKD + hypertension, management appropriate	0 (0-1)	0 (0-1)	0 (0-1)	1 (1-2)	1 (1-2)	1 (1-2)
New CKD + diabetes, management appropriate	0 (0-1)	0 (0-1)	0 (0-1)	2 (1-2)	2 (1-2)	2 (1-2)

© 2021 Tonelli M et al. *JAMA Network Open.*

Group	China			Mexico		
	Using ACE/ARB estimate from US	10% higher than US	10% lower than US	Using ACE/ARB estimate from US	10% higher than US	10% lower than US
Known CKD, management appropriate	1 (0-1)	1 (0-1)	1 (0-1)	0 (0-1)	0 (0-1)	0 (0-1)
Total CKD, but no change in management	1 (0-1)	1 (0-1)	1 (0-1)	3 (2-3)	3 (2-3)	3 (2-3)
**Need for change in management is apparent without assessing eGFR or albuminuria**						
New CKD + hypertension, management will change	1 (1-2)	1 (1-2)	1 (1-2)	6 (5-7)	6 (5-7)	6 (5-7)
New CKD + diabetes, management will change	1 (1-2)	1 (1-2)	1 (1-2)	8 (7-9)	8 (7-9)	8 (7-9)
Known CKD, management will change	1 (1-2)	1 (1-2)	1 (1-2)	0 (0-1)	0 (0-1)	0 (0-1)
Total need for change in management is apparent without assessing eGFR or albuminuria	3 (3-4)	3 (3-4)	3 (3-4)	14 (13-15)	14 (13-15)	14 (13-15)
**Need for change in management based on assessing eGFR and/or albuminuria directly, total**						
New CKD + hypertension, controlled BP, not taking ACEI/ARB, management will change	0 (0-1)	0 (0-1)	0 (0-1)	0 (0-1)	0 (0-1)	0 (0-1)
New CKD + diabetes, controlled BP/HbA1C/FPG, not taking ACEI/ARB, management will change	0 (0-1)	0 (0-1)	0 (0-1)	1 (0-1)	1 (1-2)	1 (0-1)
New CKD, no hypertension or diabetes, management will change	1 (0-1)	1 (1-2)	1 (1-2)	2 (2-3)	2 (2-3)	2 (2-3)
Total need for change in management based on assessing eGFR and/or albuminuria directly	1 (1-2)	1 (1-2)	1 (1-2)	3 (3-4)	3 (3-4)	3 (3-4)
Number needed to screen	891 (702-1,219)	1,007 (783-1,410)	970 (758-1,350)	324 (280-383)	297 (258-349)	279 (243-326)

CKD was defined as eGFR<60 ml/min/1.73m^2. Data are expressed per 1000 (95% confidence intervals).

CKD – chronic kidney disease, eGFR – estimated glomerular filtration rate, BP – blood pressure, ACEI/ARB – angiotensin converting enzyme inhibitor or angiotensin receptor blocker, US – United States, HbA1C – hemoglobin A1C, FPG – fasting plasma glucose.

Severe albuminuria was defined by any one of the following: Albumin-to-creatinine ratio (ACR) >300 mg/g or >30 mg/mmol; protein-to-creatinine ratio (PCR) >500 mg/g or >50 mg/mmol; dipstick urinalysis ≥2+ or greater for albumin.

New CKD + hypertension includes only people without diabetes. New CKD + diabetes includes people with or without hypertension.

© 2021 Tonelli M et al. *JAMA Network Open.*

For participants with CKD but without diabetes, the need for a management change was defined by non-use of ACEI/ARB or blood pressure above the target value of 140/90 mmHg. For participants with CKD and diabetes, the need for a management change was defined by non-use of ACEI/ARB, blood pressure above the target value of 140/90 mmHg, HbA1C ≥8% or fasting glucose ≥9.9 mmol/l.

Results for the number needed to screen as presented in the text cannot be directly calculated from the data in this table because of rounding.

© 2021 Tonelli M et al. *JAMA Network Open.*

eTable 3. Testing Requirements and Yield of Screening vs Case Finding Using Primary Chronic Kidney Disease Definition in China and Mexico When Drug Use Varied vs the US

Strategy	China			Mexico		
	Using ACE/ARB estimate from US	10% higher than US	10% lower than US	Using ACE/ARB estimate from US	10% higher than US	10% lower than US
Screening strategy						
N in whom measuring eGFR would be required	47,204	47,204	47,204	51,137	51,137	51,137
Identified cases of CKD in which any management change is required, N	1,065	1,073	1,058	4,701	4,735	4,667
Identified cases of CKD in which any management change is required, %	2.3 (2.1-2.4)	2.3 (2.1-2.4)	2.2 (2.1-2.4)	9.2 (8.9-9.4)	9.3 (9.0-9.5)	9.1 (8.9-9.4)
Change is required because of eGFR measurement per se, N	403	408	399	1,286	1,320	1,252
Change is required because of eGFR measurement *per se*, %	0.9 (0.8-0.9)	0.9 (0.8-0.9)	0.8 (0.8-0.9)	2.5 (2.4-2.7)	2.6 (2.4-2.7)	2.4 (2.3-2.6)
Case-finding strategy						
N in whom measuring eGFR would be required	19,234	19,234	19,234	31,489	31,489	31,489
Identified cases of CKD in which any management change is required, N	704	712	697	3,753	3,787	3,719
Identified cases of CKD in which any management change is required, %	3.7 (3.4-3.9)	3.7 (3.4-4.0)	3.6 (3.4-3.9)	11.9 (11.5-12.3)	12.0 (11.7-12.4)	11.8 (11.5-12.2)
Implications of case-finding vs screening						
% reduction in number of people recommended eGFR measurements	59.3 (58.8-59.7)	59.3 (58.8-59.7)	59.3 (58.8-59.7)	38.4 (38.0-38.8)	38.4 (38.0-38.8)	38.4 (38.0-38.8)
% increase in proportion of detected cases that require management change	62.2 (59.3-65.1)	62.9 (60.0-65.8)	61.7 (58.7-64.6)	29.6 (28.3-31.0)	29.9 (28.6-31.2)	29.4 (28.1-30.7)
Proportion of all CKD cases identified with case-finding strategy						
	66.1 (65.9-66.3)	66.4 (66.1-66.6)	65.9 (65.6-66.1)	79.8 (79.8-79.9)	80.0 (79.9-80.0)	79.7 (79.6-79.7)
Percent with management change who require eGFR to be detected						
	37.8 (37.5-38.1)	38.0 (37.7-38.3)	37.7 (37.4-38.0)	27.4 (27.3-27.4)	27.9 (27.8-28.0)	26.8 (26.7-26.9)

CKD was defined as eGFR<60 ml/min/1.73m^2. Data are expressed as percentages (95% confidence interval).

© 2021 Tonelli M et al. *JAMA Network Open.*

eGFR – estimated glomerular filtration rate, CKD – chronic kidney disease, HbA1C – hemoglobin A1C, ACEI/ARB – angiotensin converting enzyme inhibitor or angiotensin receptor blocker.

Screening was defined by measuring eGFR in all adults from the target population. Case-finding was defined by measuring eGFR only in adults with a history of hypertension, diabetes or CKD as well as those with blood pressure ≥140/90 and those with laboratory evidence of diabetes (HbA1C ≥6.5 or fasting blood glucose >7.0 mmol/l [>126 mg/dl]).

For participants with CKD but without diabetes, the need for a management change was defined by non-use of ACEI/ARB or blood pressure above the target value of 140/90 mmHg. For participants with CKD and diabetes, the need for a management change was defined by non-use of ACEI/ARB, blood pressure above the target value of 140/90 mmHg, HbA1C ≥8% or fasting glucose ≥9.9 mmol/l.

Results as presented in the text cannot be directly calculated from the data in this table because of rounding.

© 2021 Tonelli M et al. *JAMA Network Open.*

eReferences.

1. Pickering TG, Hall JE, Appel LJ, Falkner BE, Graves J, Hill MN, et al. Recommendations for blood pressure measurement in humans and experimental animals: Part 1: blood pressure measurement in humans: a statement for professionals from the Subcommittee of Professional and Public Education of the American Heart Association Council on High Blood Pressure Research. Hypertension. 2005;45(1):142-61.

2. Levey AS, Stevens LA, Schmid CH, Zhang YL, Castro AF, 3rd, Feldman HI, et al. A new equation to estimate glomerular filtration rate. Ann Intern Med. 2009;150(9):604-12.

© 2021 Tonelli M et al. *JAMA Network Open.*
